# Development and validation of a chromatographic method for determining Clematichinenoside AR and related impurities

**DOI:** 10.1186/1752-153X-6-150

**Published:** 2012-12-08

**Authors:** Yang Zhou, Yue Guan, Ji Shi, Xiaolin Zhang, Lan Yao, Lifang Liu

**Affiliations:** 1 The State Key Laboratory of Natural Medicines, China Pharmaceutical University, Nanjing 210009, China

**Keywords:** Clematichinenoside AR, Related impurities, HPLC-UV, Correction factors

## Abstract

**Background:**

Clematichinenoside AR is a promising lead compound for the treatment of rheumatoid arthritis. A systematic research for the related impurities in AR bulk samples is still lacking. For the safe use of this natural product in future clinical practice, the structure and content of each constituent, including the main ingredient as well as the impurities in AR bulk sample must be characterized in detail.

**Results:**

A simple and stability indicating RP-HPLC method was developed and validated for determining the purity of clematichinenoside AR (AR), a natural product from the roots of *Clematis manshurica* Rupr. (Ranunculaceae) with the potential of treating rheumatoid arthritis. Five impurities were characterized, and impurity 2 (Clematomandshurica saponin F) is a new triterpenoid saponin isolated from this product. Optimum separation for clematichinenoside AR and five related impurities was carried out on an Agilent octadecylsilane bonded silica gel column (TC-C18, 4.6 mm ×150 mm, 5 μm) using a gradient HPLC method. The validation results showed good sensitivity, specificity, linearity(r^2^>0.9992) precision(RSD<1.63%), accuracy(recoveries in the range of 95.60%-104.76%) and robustness. Three AR bulk samples containing all the impurities were examined by two methods, and the stability of correction factors for the determination of related impurities was discussed. The proposed stability-indicating method was suitable for the quality control of this natural product.

**Conclusion:**

Five related impurities of clematichinenoside AR were characterized, including a new triterpenoid saponins firstly found in clematichinenoside AR bulk samples. In the simple chromatographic method for determining clematichinenoside AR and its related impurities in bulk samples, the correction factor was better for the quality control in the relative stable concentrations.

## Background

Radix et Rhizoma Clematidis (Wei-Ling-Xian) has been used as an anti-inflammatory, antitumor, and analgesic agent in traditional Chinese medicine (TCM) with a long history [1,2]. Triterpenoid saponins are considered to be the main bioactive constituents in the root extracts of clematis species, and recent pharmacological studies revealed that some triterpenoid saponins have significant anti-inflammatory, antitumor and analgesic activities [3-7]. Clematichinenoside AR, 3-O-β-[(O-α-L-rhamnopyranosyl-(1→6)-O-β-D-glucopyranosyl-(1→4)-O-β-D-glu-copyranosyl-(1→4)-O-β-D-ribopyranosyl-(1→3)-α-O-L-rhamnopyranosyl-(1→2)- α-L-arabinopyranosyl)oxy] oleanolic acid 28-O-α-L-rhamnopyranosyl-(1→4)- β-D-glucopyranosyl-(1→6)-β-D-glucopyranosyl ester, is a typical triterpenoid saponin isolated from the roots of *Clematis chinensis* Osbeck and *Clematis manshurica* Rupr. (Ranunculaceae). According to previous pharmacological studies, clematichinenoside AR has a potential anti-inflammatory effect in arthritic rats, and its mechanism may involve the inhibition of the expression of NF-κB p65 subunits, TNF-a and COX-2 [8-11]. In previous study, we established a HPLC-ELSD method to analyze triterpenoid saponins from Chinese clematis [12] and a LC-MS/MS method to detect clematichinenoside AR in rats after oral administration [13]. Our previous work revealed that clematichinenoside AR is a promising lead compound for the treatment of rheumatoid arthritis. For the safe use of this natural product in future clinical practice, the structure and content of each constituent, including the main ingredient as well as the impurities in AR bulk sample must be characterized in detail, as required by the State Food and Drug Administration (SFDA) in China. For this purpose, AR bulk samples were prepared using industry-scale production procedures with the AR content higher than 90% (quantified by HPLC). Five related impurities were observed in chromatography, and the structure of the actual impurities that exceed 0.1% was characterized [14]. Besides, a new compound, clematomandshurica saponin F was isolated for the first time and confirmed as a new triterpenoid saponin by spectral methods (MS, ^1^H NMR, ^13^C NMR, COSY, DEPT, HMBC, HSQC and ROESY).

### Experimental

#### Materials and reagents

All the chemical reference substances, including clematichinenoside AR and related substances were prepared in our laboratory, and the purity of each compound was confirmed to be higher than 98% by HPLC. Three batches of AR bulk samples were supplied by Chia Tai Tianqing Pharmaceutical Company. HPLC grade acetonitrile were purchased from Merck (Darmstadt, Germany), other analytical grade reagents were purchased from Nanjing Chemical Reagent Co., Ltd. (Nanjing, China).

#### Apparatus and chromatographic conditions

Analyses were primarily performed on an Agilent 1200 HPLC system (Agilent Technologies, CA, USA), equipped with a variable wavelength detector (VWD). The HPLC separation method was developed on an Agilent reversed phase octyldecyl silica column (TC-C18, 4.6 mm × 150 mm, 5 μm). The mobile phase consisted of solvent A(water) and solvent B(acetonitrile) with the following gradient program: 30% B in 0~5 min; 30%~35% B in 5~12 min; 35%~60% B in 12~17 min; 60% B in 17~20 min. (The flow rate was set at 1.0 mL/min, column temperature was kept at 30°C with the UV detection at 203 nm and the injection volume was 20 μL).

#### Isolation and purification of related impurities

For the isolation, a glass column with ODS material and semi-preparative HPLC instrument using an Agilent 1200 with Agilent Eclipse XDB-C18 (9.4×250 mm, 5 μm, USA) were used to enrich and purify impurities. AR bulk sample (Lot: 20110610) dissolved in water was subjected to ODS column chromatography and eluted with gradient water–methanol. Each collected fraction was analyzed by TLC and HPLC. The fractions of the same compound were pooled together and purified by HPLC instrument. These impurities were used as reference substances in the following studies after structural elucidation by TOF-MS and NMR.

#### Preparation of standard solutions

AR reference standard solution (2.0 mg/mL) and five impurity solutions (1.0 mg/mL) were prepared respectively in a mixture of acetonitrile and water (30:70, v/v) as standard stock solutions. The mixed standard solution was obtained by transferring the appropriate volumes of every stock solution into a 5 mL volumetric flask and diluting the mixture with the mobile phase in order to get standard solutions containing 1.0 mg/mL AR and 0.05mg/mL of each impurity (at 5.0% concentration relative to AR).

## Results and discussion

### Structure elucidation of related impurities

#### Structure elucidation of impurity 2

Impurity 2 (Clematomandshurica saponin F) was a white, amorphous powder, and was freely dissolved in water and methanol, mp 231-232°C. The optical rotation([a]^20^_D_) was −38.1 (c 0.10, 30% acetonitrile). The IR spectrum showed the presence of hydroxyl groups at 3425 cm^-1^ and carbonyl groups at 1640 cm^-1^.

HR-ESI MS gave a strong molecular ion with the m/z value of 1821.8175, suggesting the molecular formula as C_82_H_134_O_44_. The NMR spectra were recorded at 303k on a Bruker AV-500 NMR (1H NMR, 500 MHz; 13C NMR, 125 MHz) instrument using pyridine-d5.

The ^13^C NMR spectral data reported in Table 1 are in agreement with those in the literature [15]. More details of the structure were elucidated by analyzing the ^1^H and ^13^C NMR spectra. The ^13^C NMR spectrum showed 82 carbon signals. There were 28 signals at upfield (δ 0–60), in which 25 signals were due to aglycone and three signals from C-6 of the three rhamnose (δ 18.4, 18.5, 18.6). 39 signals were shown from δ 60 to δ 90, in which δ 81.9 and δ 63.9 were from the aglycone. 9 signals exhibited from δ 90 to δ 100 (δ 95.6, 101.4, 102.7, 102.7, 103.2, 104.6, 104.7, 104.8, 104.9) were signals of the sugar anomeric carbons. There were two signals showed from δ 110 to δ 150, which were from the aglycone. The signal at δ 176.5 was due to carbonyl group. The ^13^C NMR spectral data reported in Table 1 are in agreement with those in the literature [15], suggesting the hederagenin as the aglycone. The ^1^H NMR spectrum exhibited nine signals of the sugar anomeric protons at δ 5.04 (1H, m, H-1 of Ara), 6.25 (1H, brs, H-1 of Rha), 5.80(1H, brs, H-1 of Rib), 4.91(1H, m, H-1 of Glc), 5.07(1H, d, H-1 of Glc’), 5.40(1H, brs, H-1 of Rha’), 6.20(1H, d, H-1 of Glc”), 4.97(1H, d, H-1 of Glc”’), 5.81(1H, s, H-1 of Rha”) and methyl signals of the three rhamnose at δ 1.67(3H, d, J=6.1 Hz, Me-6 of Rha”), 1.57(3H, d, J=6.0Hz, Me-6 of Rha’), 1.51(3H, d, J=6.0 Hz, Me-6 of Rha). Also there are six methyl signals of aglycone shown in the ^1^H NMR spectrum at δ 1.15(3H, s, Me-27), 1.10(3H, s, Me-24), 1.06(3H, s, Me-26), 0.94(3H, s, Me-25), 0.87(3H, s, Me-30), 0.85(3H, s, Me-29). The downfield shift of C-3 and the upfield shift of C-28 suggested that the hydroxyl group of C-3 and the carbonyl group of C-28 were both glycosydated. The chemical shift of C-23 moved to the downfield at δ 63.96 because of the hydroxylation. The acid hydrolysis showed the presence of arabinose, glucose, rhamnose and ribose. Compared with the data in the literature [5], the exact sugar sequence and its linkage position to the aglycone were solved by a detailed analysis of the 2D NMR spectra. The HMBC spectrum showed the correlations from H-Rha’-1 to C-Glc’-6, H-Ara-1 to C-3; H-Ara-2 to C-Rha-1; H-Rha-1 to C-Ara-2; H-Rha-3 to C-Rib-1; H-Glc-1 to C-Rib-4; H-Glc’-1 to C-Glc-4; H-Glc”-1 to C-28; H-Glc”’-1 to C-Glc”-6; H-Rha”-1 to C-Glc”’-4. Based on these analyses, the structure of this compound was elucidated as 3-O-β-[(O-α-L-rhamnopyranosyl-(1→6)-O-β-D-glucopyranosyl-(1→4)-O-β-D-glucopyranosyl-(1→4)-O-β-D-ribopyranosyl-(1→3)-O-α-L–rhamnopyranosyl-(1→2) -α-L-arabinopyranosyl)oxy] hederagenin 28-O-α-L- rhamnopyranosyl-(1→4)-O-β-D- glucopyanosyl-(1→6)- β-D-glucopyanosyl ester. The spectra data assignments were shown in Table 1.

**Table 1:** ^**1**^**H and **^**13**^**C NMR spectra data of clematomandshurica saponin F (pyridine-*d*5)**

**Position**	**δ**_**C**_**/ppm**	**δ**_**H**_**/ppm**
1	39.13	1.06,1,51
2	26.39	1.99,2.20
3	81.93	4.13
4	43.62	
5	47.73	1.67
6	18.17	1.26,1.28
7	32.78	1.51,1.52
8	39.94	
9	48.24	1.71
10	36.92	
11	23.39	1.88
12	123.72	5.37
13	144.12	
14	42.15	
15	28.31	2.20
16	23.85	1.99
17	47.05	
18	41.67	3.12,3.15
19	46.22	1.19,1.67
20	30.74	
21	34.03	1.28
22	32.57	1.70,1.85
23	63.96	4.24,3.89,CH_2_OH
24	14.09	1.10,s
25	16.21	0.94,s
26	17.56	1.06,s
27	26.07	1.15,s
28	176.51	
29	33.11	0.85,s
30	23.72	0.87,s
	3-o-sugar	
Ara 1	104.95	5.04
2	75.18	4.52
3	74.77	4.24
4	69.28	4.28
5	66.25	3.65,4.22
Rha 1	101.41	6.25,brs
2	71.95	4.82
3	82.02	4.67
4	72.77	4.37
5	69.87	4.63
6	18.45	1.51,d,6.0Hz
Rib 1	104.63	5.80,brs
2	72.64	4.38
3	69.71	4.54
4	76.55	3.85
5	61.97	4.40
Glc 1	103.22	4.91,m
2	74.14	4.28
3	76.69	4.28
4	81.05	4.63
5	75.36	3.91
6	61.75	4.20
Glc^’^ 1	104.85	5.07,d,8.1Hz
2	74.24	3.83
3	78.37	4.16
4	71.95	4.70
5	76.80	4.02
6	68.57	3.92,4.60
Rha^’^ 1	102.74	5.40,brs
2	71.75	4.68
3	72.56	4.52
4	73.89	4.18
5	69.75	4.27
6	18.52	1.57,d,6.0Hz
	28-o-sugar	
Glc^”^ 1	95.65	6.20,d,8.0Hz
2	73.83	4.07
3	78.75	4.14
4	70.95	4.24
5	78.05	4.07
6	69.28	4.62
Glc^”’^ 1	104.71	4.97,d,7.8Hz
2	74.85	3.88
3	76.40	3.85
4	78.22	4.38
5	77.16	3.63
6	61.35	4.07
Rha^”^ 1	102.76	5.81
2	72.56	4.07
3	72.83	4.50
4	74.09	3.95
5	70.32	4.91
6	18.62	1.67,d,6.1Hz

#### Structure elucidation of impurities 1, 3, 4, 5

**Figure 1:**
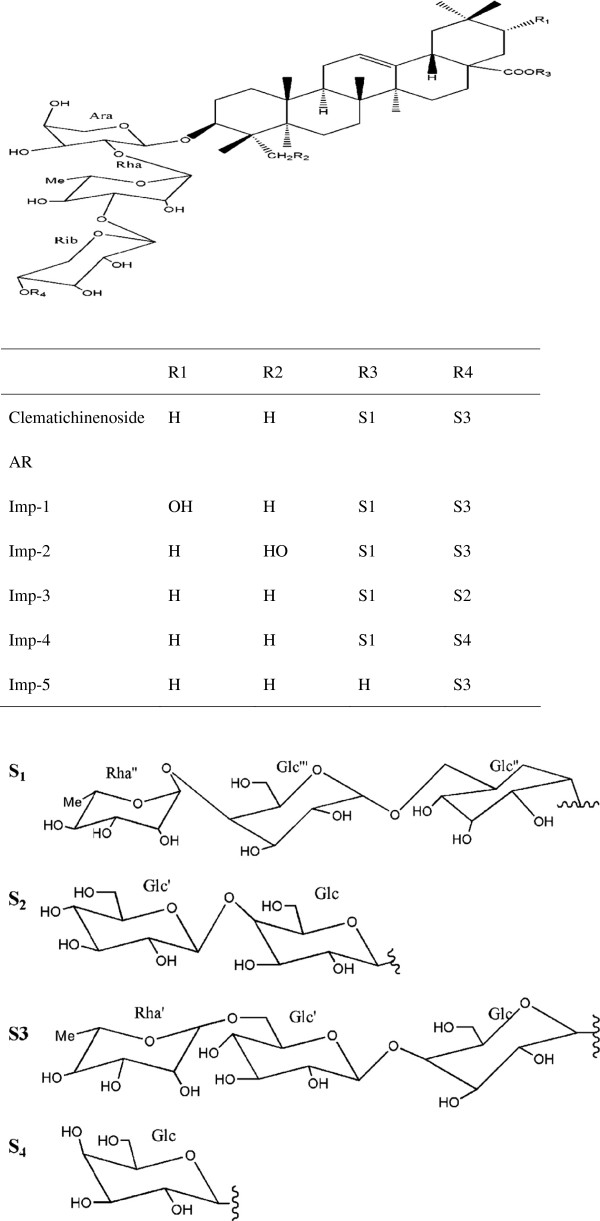
The chemical structures of Clematichinenoside AR and its related impurities.

Impurities 1, 3, 4, 5 were obtained as white, amorphous powders. The TOF-MS revealed impurities 1, 3, 4 have double charged molecular ion at m/z 933.4001, 852.3874, 794.3646, and gave impurity 5 has molecular ion at m/z 1335.6913, which corresponds to a molecular formula of C_82_H_134_O_44_, C_76_H_124_O_39_, C_69_H_124_O_34_, and C_64_H_104_O_29_, respectively. Their chemical structures were suggested as clematichinenoside AR_6_[3], clematomandshurica saponin C [4], clematichinenoside C [16], and clematichinenoside AR_2_[5] by comparing their mass spectral data with those published in the literature. The structures of these related substances were further confirmed by the same retention time in HPLC chromatogram with each of their reference substances shown above. The chemical structures of these related substances are shown in Figure 1.

#### Development and validation of HPLC method

**Figure 2:**
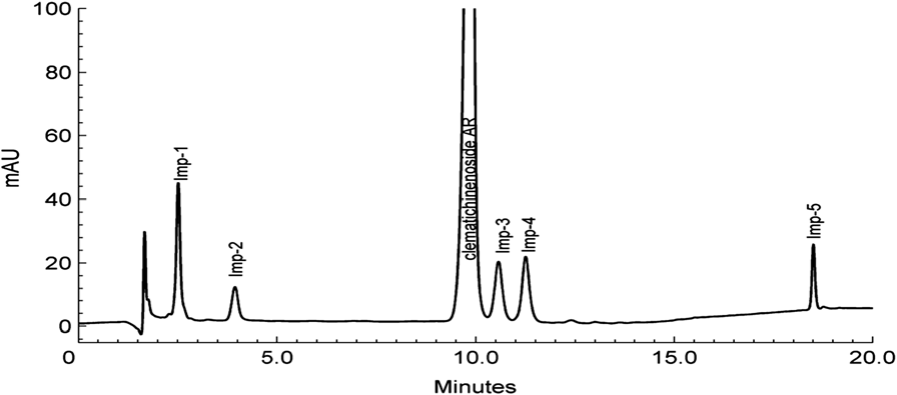
A typical chromatogram of clematichinenoside AR bulk sample.

A simple, efficient and reliable method was developed to examine AR and five impurities in bulk samples. AR and its related impurities all showed maximum absorption at 203 nm. Therefore, the detection wavelength was set at 203 nm, flow rate and column temperature were optimized in order to achieve the desired resolution and tailing factor of closely eluting impurities using simple chromatographic conditions. Under this condition, all the peaks were clearly separated. The proposed method was validated with respect to system suitability, specificity, robustness, linearity, accuracy, precision, LOD and LOQ. Figure 2 showed a typical chromatogram of AR and five potential impurities. Good peak symmetries and resolutions were observed for AR and all spiked impurity components in 20 min. Results were summarized in Table 2.

**Table 2:** Method validation data for clematichinenoside AR and its related impurities

**Parameters**	**Imp-1**	**Imp-2**	**AR**	**Imp-3**	**Imp-4**	**Imp-5**
**System suitability**						
RT^b^	2.533±0.008	3.934±0.016	9.793±0.041	10.518±0.037	11.206±0.037	18.449±0.011
RRT^b,c^	0.259±0.0003	0.402±0.0002	1.000±0.0000	1.074±0.0008	1.144±0.0012	1.884±0.0071
R_S_^b,c^		6.79±0.050	18.33±0.175	1.99±0.008	2.06±0.008	26.91±0.381
A_S_^b,c^	1.124±0.004	1.047±0.057	0.838±0.003	0.964±0.003	0.974±0.004	1.012±0.0091
α ^b, c^		1.872±0.434	1.872±0.434	1.080±0.008	1.070±0.006	1.686±0.056
K’ ^b, c^	0.693±0.005	1.630±0.011	5.547±0.028	6.032±0.025	6.492±0.025	11.335±0.007
NTP ^b, c^	4009±44	3889±107	10299±174	14992±321	19418±130	248207±2222
Linearity						
r^2^	0.9998	0.9994	0.9994	0.9998	0.9994	0.9992
Slope	2.9525	2.9072	2.9442	3.1068	3.5708	4.9601
Intercept	−0.0324	−2.3100	0.2528	−0.9281	−2.0499	0.3703
Precision(RSD n=6,,%)	0.76	1.04	0.22	0.76	0.45	1.63
LOD(μg·ml-1)	0.55	1.87	0.49	0.57	0.53	1.04
LOQ(μg·ml-1)	1.10	2.80	0. 99	1.13	1.06	2.08
Accuracy (80%)	99.88%	99.54%	98.73%	100.29%	95.60%	104.76%
Accuracy (100%)	99.30%	100.36%	99.44%	100.23%	99.56%	96.15%
Accuracy(120%)	101.23%	96.50%	103.56%	102.67%	98.08%	98.08%

*RT*, retention time/min; *RRT*, relative retention time; *R*, resolution; *A*, tailing factor; *a*, selectivity; *K’*, capacity; *NTP*, number of theoretical plates. ^b^Average of five determinations .^c^Mean±SD (n = 5).

#### Effect of three C18 columns

To investigate the effect of the HPLC column on the separation and tailing factor under the optimized conditions, octyldecyl silica gel columns from three different vendors were evaluated for the analysis of AR and its impurities. These HPLC columns included: (1) Thermo ODS Hypersil (4.6 mm × 150 mm, 5 μm, USA); (2)Diamonsil C18(4.6 mm × 150 mm, 5 μm, China) (3)Agilent TC-C18(4.6mm × 150mm, 5 μm, USA). Based on the results from the laboratory HPLC system, all the resolutions were more than 1.50, but the Agilent TC-C18 provided the best resolutions. The tailing factors of AR and impurities obtained on the Agilent TC-18 column were between 0.8 and 1.2, which indicated good peak shapes for all compounds. Tailing factors of Imp-1, 5 on the Diamonsil C18 column were less than 0.8 and the numbers of theoretical plates for Imp-2, 3, 4 and AR were less than those obtained on the Agilent TC-C18. Besides, the tailing factors of Imp-1, 5 were bigger than 1.5 and was not suitable for tailing factor requirement on the Thermo ODS Hypersil. Thus, the Agilent TC-C18 column demonstrated the desirable performance and was selected for further studies. The results were listed in Table 3.

**Table 3:** Comparison of three columns under optimized conditions

**Columns**	**Compounds**	**Rs**	**As**	**α**	**k’**	**NTP**
Thermo ODS Hypersil	Imp-1	-	1.681	-	0.279	8610
(4.6mm ×150mm, 5 μm)	Imp −2	3.62	1.281	1.234	0.579	3280
	AR	9.49	0.879	2.583	2.178	3162
	Imp-3	1.60	1.036	1.119	2.559	3255
	Imp-4	1.63	0.934	1.116	2.979	3615
	Imp-5	40.03	2.778	3.215	10.974	127412
Diamonsil C18(4.6mm ×150mm, 5 μm)	Imp-1	-	0.707	-	0.237	5722
	Imp-2	3.37	1.135	1.283	0.586	2009
	AR	9.41	0.878	3.051	2.786	2167
	Imp-3	1.52	0.977	1.135	3.297	2448
	Imp-4	1.90	0.951	1.157	3.977	2948
	Imp-5	27.88	0.691	2.341	10.740	159346
Agilent TC-C18(4.6mm×150mm,5 μm)	Imp-1	-	1.124	-	0.693	4009
	Imp-2	6.79	1.047	1.872	1.630	3889
	AR	18.33	0.838	2.856	5.547	10299
	Imp-3	1.99	0.964	1.080	6.032	14992
	Imp-4	2.06	0.974	1.070	6.492	19418
	Imp-5	26.91	1.012	1.686	11.335	248207

*Rs*, resolution; *As*, tailing factor; *α*, selectivity; *k’*, capacity factor; *NTP*, number of theoretical plates.

#### System suitability

The system suitability tests were conducted using the working solution containing AR (1.0 mg/mL) and five impurities (each at 0.05 mg/mL) by five repeated injections with the optimized method. It was observed that the tailing factors for all compounds were between 0.8 and 1.2, and the resolutions were greater than 1.50. These results met the HPLC method requirements for separation and quantification of AR and related impurities in bulk samples.

#### Specificity

**Figure 3:**
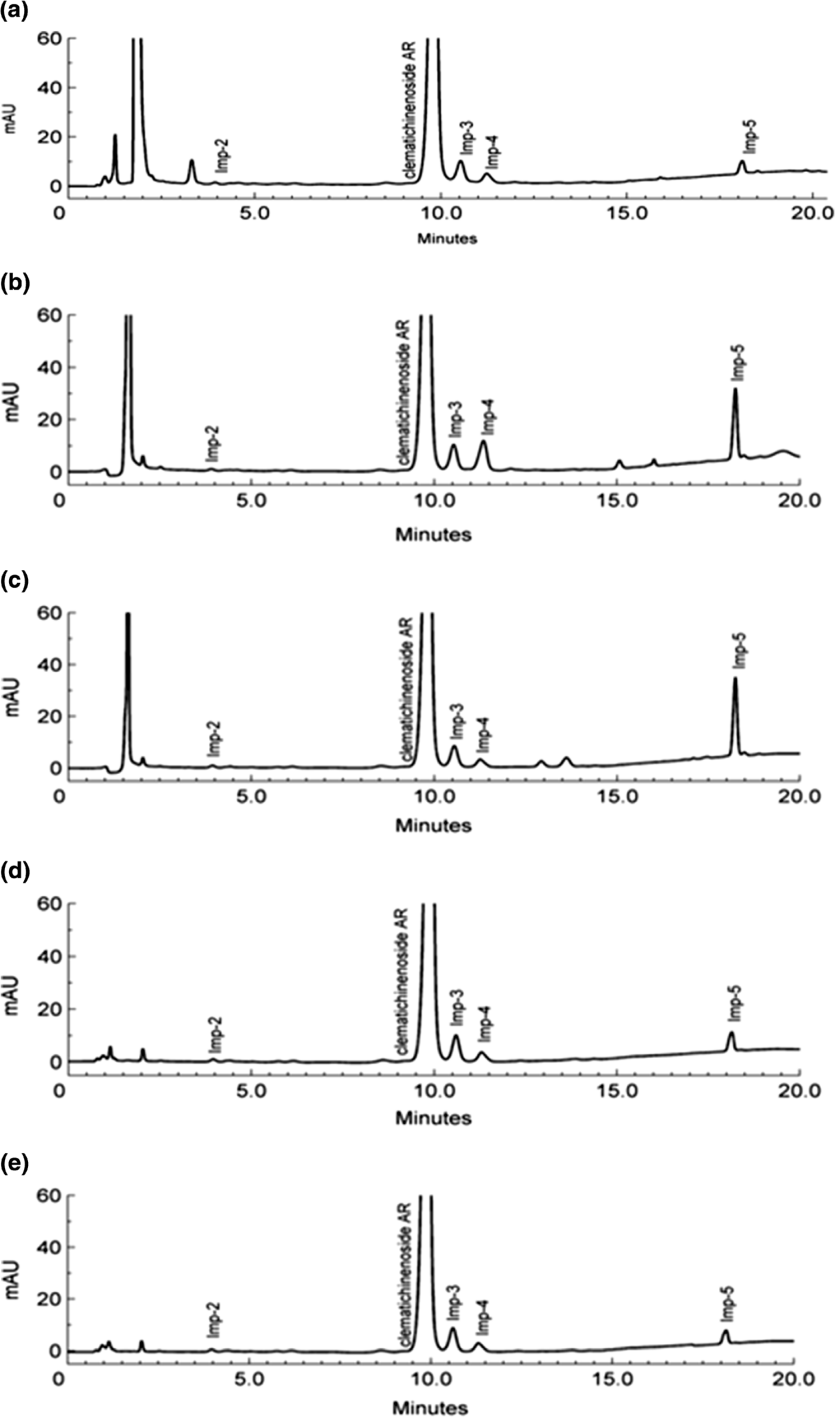
Typical chromatogram of clematichinenoside AR under stress conditions: (a) oxidative degradation, (b) acid hydrolysis, (c) base hydrolysis, (d) thermal degradation, (e) photolytic degradation.

The specificity of the related compounds and AR was assessed using the forced degradation studies under conditions described in the literature [17]. Basic, acidic, oxidative, photolytic and thermal degradation were conducted in 0.1 N NaOH at 60°C for 40 min, 1N HCl at 60°C for 30 min, 3% H_2_O_2_ at 60°C for 1 h, illumination of 1.2 million lux hours for 10 days and heat at 60°C for 8 h, respectively. Photolytic and thermal studies were conducted by exposing the samples both in solution (1.0 mg/mL) and in solid state. Samples were withdrawn at the appropriate times, the pH was adjusted to neutral and the samples were subjected to HPLC analysis after suitable dilution (1.0 mg/mL) to evaluate the ability of the proposed method to separate AR from its potential impurities. The bulk drug and degradation samples were examined by peak purity testing utilizing DAD detector. The purity factor obtained from AR peak was higher than threshold, which demonstrated the spectral homogeneity. Degradation of the sample at 60°C and illumination of 1.2 million lux hours did not show any difference in terms of chromatographic behavior compared to a fresh one, but in the acid and basic conditions, the content of Imp-5 increased significantly. Besides, clematichinenoside AR was transformed into Imp-4 in acidic condition. All the results demonstrated that degradation products formed during the stress studies were well separated from AR which proved that the adopted method was specific (Figure 3).

#### Precision

The method reproducibility was evaluated by determining precision on a spiked AR solution (1.0 mg/mL) with each impurity at 5.0% concentration (0.05 mg/mL). Each RSD (%) of peak areas for all components was below 2.0% as shown in Table 2, suggesting good method reproducibility.

#### LOD and LOQ

The standard solutions containing both AR and impurities were injected and the peak height of each component was recorded. The limit of detection (LOD) and limit of quantitation (LOQ) for AR and five impurities were calculated by normalization of the signal/noise ratio (S/N) to 3 for LOD and S/N to 10 for LOQ, see Table 2. The RSD for LOQ concentrations for all compounds was below 5.0%.

#### Linearity

The linearity of test solution was prepared at six concentration levels, 0.01 to 2.0 mg/mL for AR, from 6 to 60 μg·ml^-1^ for impurity 2 and 1, from 10 to 100 μg·ml^-1^ for impurities 3, 4 and 5. The adopted method demonstrated good linearity for clematichinenoside AR (r^2^>0.9994) and five impurity components (r^2^>0.9992).

#### Accuracy

The accuracy of the method was checked for three known concentration levels (80%, 100%, 120%) added to a sample containing all compounds and peak area was recorded. The mean analyte recoveries and their recovery rates obtained for each level are described in detail in Table 2. The accuracy and recovery results were suitable for the quantitative HPLC method.

#### Robustness

For an analytical method to be robust, it must be able to produce quantitative results despite small changes in the experimental parameters, which may occur in a typical testing laboratory. The robustness of the method was studied by employing an experimental design, where the temperature of the column (25°C −35°C) and the flow rate of the mobile phase (0.8-1.2mL·min^-1^) were purposely subjected to small modifications. The temperature, the flow rate and the combined effects as the three factors were studied on the characteristics of the chromatogram of AR and its impurities. The tailing factors for all compounds were between 0.8 and 1.2 and the resolutions were greater than 1.50.

### Application of correction factors

#### Calculation of correction factor

Due to the difficulties and expenses to prepare all the related reference standards, the application of the external standard method was limited, so correction factor was used in the quantification of related substances. According to ICH guiding principles, if the response factors of drug substance and relevant impurity are not close, we can use the drug substance as a standard to estimate the levels of impurities with providing a correction factor. The method of calculating correction factor is the same as conversion factor [18]. The correction factor (f) was the ratio of responses between internal standard substance (As/Cs) and standard substance(A_R_/C_R_).

(1) $$f=\;\frac{A_{s}/C_{s}}{A_{R/}C_{R}}\;$$

As: peak area of internal standard substance; Cs: concentration of internal standard substance; A_R:_ peak area of standard substance; Cs: concentration of standard substance

In this study, AR was selected as internal standard, and each related impurity was as standard. The correction factor of each substance was obtained as the mean values calculated from five different concentrations. The results from five gradient concentrations showed that the correction factors of Imp-1 (F1) was 1.00 ± 0.04, the Imp-2 (F2) was 1.13 ± 0.02, the Imp-3 (F3) was 1.00 ± 0.02, the Imp-4 (F4) was 0.90 ± 0.04,and the Imp-5 (F5) was 0.61 ± 0.02. The RSDs for corrections for all compounds were below 5%. Additionally, there were other two approaches used to calculate the correction factors. The first one was calculated by the ratio of equation slope of AR and its impurities. F1 was 1.00; F2 was 1.01; F3 was 0.95; F4 was 0.82 and F5 was 0.59. The above results showed that F1 and F5 were similar while the others had significant difference. The linearity data showed that the interception of impurities 2 and 4 were not less than 1. This indicated that we couldn't neglect the influence of interception. The second one was only by one single concentration solution. But Table 4 clearly showed that the value of F1 increased with the increase of concentration, the change of F5 was exact contrary. F2 was more stable when the concentration was above 10 μg/mL. The reason may relate with the extremely low absorption value of saponins. When the concentration of analytes was lower than 10 μg/mL, the RSD would increase. Thus, we suggest that the correction factor should be applied in a relative stable range of concentrations, if the concentration of impurity 2 was too low in the bulk sample, the external standard method would be desirable.

**Table 4:** Correction factors of related impurities

	**Imp-1(μg/mL)**	**F1**	**Imp-2(μg/mL)**	**F2**	**AR(μg/mL)**	**F0**	**Imp-3(μg/mL)**	**F3**	**Imp-4(μg/mL)**	**F4**	**Imp-5(μg/mL)**	**F5**
1	6.41	0.96	6.26	1.15	9.52	1	9.83	1.01	6.50	0.94	5.98	0.60
2	12.82	0.97	12.53	1.13	19.05	1	19.66	1.02	13.01	0.91	11.95	0.60
3	26.70	1.00	26.10	1.13	39.68	1	40.96	0.99	27.10	0.89	24.90	0.63
4	40.05	1.02	39.15	1.11	59.52	1	61.44	1.00	40.65	0.89	37.35	0.61
5	53.40	1.03	52.20	1.12	79.36	1	81.92	0.99	54.20	0.89	49.80	0.60
RSD(%)		3.06		1.31				1.30		2.42		2.14
Average		1.00		1.13				1.00		0.90		0.61

#### Ruggedness and robustness

In the quality control of this natural product, the value of correction factor may change greatly if it was performed in different laboratories. Therefore, the stability of correction factor was investigated by ruggedness and robustness tests. In ruggedness test, five columns (three different Agilent TC-18 columns, Thermo ODS Hypersil and Diamonsil C18) were investigated. All these columns meet with the requirements of system suitability parameters which was shown in Table 3. In robustness tests, three related factors were studied, the temperature of the column (25°C-35°C), the flow rate of the mobile phase (0.8-1.2 mL·min^-1^) and the UV detection wavelength (200 nm-206 nm). The results were shown in Table 5. On all Agilent TC-C18 columns, the mean correction factor of five impurities was 1.00, 1.12, 1.00, 0.90 and 0.62, respectively. The result revealed that all the correction factors were stable on different Agilent TC-C18 columns. Besides, the value of F3 and F4 were stable when it was performed in different brands of chromatographic columns and modified experimental parameters, the reason may be the structure of Imp – 3 and Imp - 4 was very close to AR. Factors regarding the flow rate and column temperature have some influence on the Imp-2. When the flow rate was decreased from 1.2 to 0.8, the correction factor of Imp-2 was increased from 1.10 to 1.16. Regarding column temperature, when the temperature was increased from 25°C to 35°C, the correction factor of Imp-2 was decreased from 1.16 to 1.1. The possible reason may relate with the integration parameters, the peak area changed at the same integration parameters when there were small changes in experimental parameters.

**Table 5:** Results of ruggedness and robustness test

	**Imp-1**	**Imp-2**	**Imp-3**	**Imp-4**	**Imp-5**
**Diamonsil**	0.98	1.02	0.92	0.80	0.58
**Thermo**	1.08	1.12	1	0.9	0.77
**206 nm**	1.03	1.11	0.99	0.89	0.66
**200 nm**	1.02	1.07	0.99	0.89	0.72
**35°C**	1.04	1.1	0.99	0.89	0.68
**25°C**	1.02	1.16	0.99	0.89	0.71
**1.2 ml/min**	0.99	1.10	0.98	0.88	0.68
**0.8 ml/min**	1.03	1.16	0.99	0.90	0.67
**TC-C18 **	0.99	1.12	1.00	0.90	0.63
**TC-C18 **	1.02	1.12	0.99	0.90	0.62
**TC-C18 **	1.00	1.13	1.00	0.90	0.61

#### Analysis of three batches of clematichinenoside AR bulk samples

The validated method was applied to quantitate impurities in three batches of AR bulk samples by using two methods: external standard method as method I, and single reference standard by five correction factors were used to determine the impurities of the same samples as method II. The sample solutions were prepared at 1 mg/mL. The contents of impurities relative to AR were summarized in Table 6. The RSDs of sum contents of the five compound obtained by the two methods of each sample were less than 2%. And the RSDs of Imp-1, Imp- 4, Imp-5 were more than 5%. The reason might relate with the low concentration of impurities in samples. However, the difference can be neglected for quality control because the RSDs of sum contents meet the requirements. Therefore, single reference standard by correction factors could be used in the quality control of related impurities. According to the regulation of State Food and Drug Administration, the content of AR should not be less than 90.0%. So, all the samples comply with the limit.

**Table 6:** The contents of clematichinenoside AR and its related impurities in three batches of bulk samples

	**Bulk-1**			**Bulk-2**			**Bulk-3**		
	**methodI**	**methodII**	**RSD**	**methodI**	**methodII**	**RSD**	**methodI**	**methodII**	**RSD**
Imp-1(%)	0.65	0.67	2.14	1.11	0.99	8.08	-	-	-
Imp-2(%)	-	-	-	-	-	-	2.47	2.36	3.22
clematichinenoside AR(%)	93.01	-	-	92.22	-	-	95.03	-	-
Imp-3(%)	4.38	4.57	3.00	4.17	4.34	2.83	2.08	2.12	1.35
Imp-4(%)	1.24	1.35	6.01	1.22	1.39	9.21	-	-	-
Imp-5(%)	1.02	0.89	9.63	1.03	0.94	6.46	-	-	-
Sum(%)	7.28	7.48	1.92	7.53	7.66	1.21	4.55	4.48	1.10

## Conclusions

The quality of bulk drug not only depends on the adopted procedure, but also on the side-reaction products, unreacted raw materials and intermediates, because they may render unwanted toxicological effects. Hence, thorough examination of related impurities plays a very important role in controlling the quality of bulk drug in the final product [14,19]. The impurities were isolated from AR bulk samples by column chromatography and semi-preparative HPLC methods. Five related impurities were characterized, including a new compound confirmed as 3-O-β-[(O-α-L-rhamnopyranosyl-(1→6)-O-β-D-glucopyranosyl-(1→4)-O-β-D -glucopyranosyl-(1→4)-O-β-D-ribopyra-nosyl-(1→3)-O-α-L–rhamnopyranosyl-(1→2) -α-L-arabinopyranosyl)oxy] hederagenin 28-O-α-L- rhamnopyranosyl-(1→4)-O-β-D-glucopyanosyl -(1→6)-β-D- glucopyanosyl ester. Imp-1,3,4,5 were four known triterpenoid saponins isolated from Rhizoma Clematidis(Wei-Ling-Xian), Imp-2 (clematomandshurica saponin F) was a new triterpenoid saponins firstly found in AR bulk samples, it might be derived from the crude drug. In this paper, a simple gradient RP-HPLC method was developed and validated for the simultaneous determination of AR and its related impurities in bulk samples. By using experimental designs, optimum separation was successfully achieved and five impurities of AR could be separated in 20 minutes with satisfactory resolution, linearity, and sensitivity. Furthermore, two methods including external standard method and single reference standard with correction factor were applied to determine impurities in three batches of AR bulk samples. In order to ensure the accuracy of the determination, speed up the analysis, and reduce the cost of experimental procedure, the correction factor was better for the determination of related substances in a relative stable range of concentrations, which could be very helpful for the quality control of AR bulk sample and the safe clinical use in the future.

## Abbreviations

HPLC: High-performance liquid chromatography; ICH: International Conference on Harmonization; UV: Ultraviolet; LOD: Limit of detection; LOQ: Limit of quantification; SD: Standard deviation; RSD: Relative standard deviation; ELSD: Evaporative Light Scattering Detector; RP-HPLC: Reverse phase high-performance liquid chromatography; LC-MS/MS: Liquid chromatography-tandem mass spectrometry; TOF-MS: Time-of-flight mass spectrometry nuclear magnetic resonance; HMBC: Heteronuclear Multiple Bond Correlation; HR-ESI MS: High resolution electrospray ionization mass spectrometry; Rs: Resolution; As: Tailing factor; α: Selectivity; k’: Capacity factor; NTP: Number of theoretical plates; AR: Clematichinenoside AR.

## Competing interests

The authors declare that they have no competing interests.

## Authors’ contributions

LFL planed and supervised the whole work, YZ carried out the experiments and analyzed the data statistically, YG, JS, XLZ and LY participated in writing the manuscript. All authors read and approved the final manuscript.
